# Log in and breathe out: efficacy and cost-effectiveness of an online sleep training for teachers affected by work-related strain - study protocol for a randomized controlled trial

**DOI:** 10.1186/1745-6215-14-169

**Published:** 2013-06-11

**Authors:** Hanne Thiart, Dirk Lehr, David Daniel Ebert, Bernhard Sieland, Matthias Berking, Heleen Riper

**Affiliations:** 1 Innovation Incubator, Division of Health Training Online, Leuphana University Lueneburg, Rotenbleicher Weg 67, 21335, Lueneburg, Germany; 2 Philipps University Marburg, Philipps-Universität Marburg, Gutenbergstraße 18, 35037, Marburg, Germany; 3 Faculty of Psychology and Education, VU University Amsterdam, Van der Boechorststraat 1, 1081BT, Amsterdam, The Netherlands

**Keywords:** Insomnia, Internet, CBT-I, Cost-effectiveness, Occupational health, Stress, Teachers

## Abstract

**Background:**

Insomnia and work-related stress often co-occur. Both are associated with personal distress and diminished general functioning, as well as substantial socio-economic costs due to, for example, reduced productivity at the work place and absenteeism. Insomnia complaints by people experiencing work-related stress are correlated with a deficient cognitive detachment from work. Diffuse boundaries between work and private life can additionally complicate the use of recreational activities that facilitate cognitive detachment.

Cognitive behavioral therapy for insomnia is effective but rarely implemented. Internet-based cognitive behavioral therapy for insomnia could potentially reduce this deficit given its demonstrated effectiveness. Less is known, however, about the efficacy of internet-based cognitive behavioral therapy for insomnia in populations affected by high work stress. Thus, the aim of the present study is to evaluate the efficacy and cost-effectiveness of a newly developed, guided online training which is based on Cognitive Behavioral Therapy for insomnia and tailored to teachers affected by occupational stress.

**Methods/Design:**

In a two-arm randomized controlled trial (N = 128), the effects of a guided online sleep training will be compared to a waitlist-control condition. German teachers with significant clinical insomnia complaints (Insomnia Severity Index ≥15) and work-related rumination (Irritation Scale, subscale Cognitive Irritation ≥15) will be included in the study. The primary outcome measure will be insomnia severity. Additionally, an economic evaluation from a societal perspective will be conducted. Data from the intention-to-treat sample will be analyzed two and six months after randomization.

**Discussion:**

To the best of our knowledge, this is the first study to evaluate an online sleep training tailored to a specific population with work stress, that is, teachers. If this type of intervention is effective, it could reduce the paucity of cognitive behavioral therapy for insomnia and augment the support for teachers in coping with their insomnia problems.

**Trial registration:**

German Clinical Trial Register (DRKS): DRKS00004700

## Background

Sleep and work represent areas of life in which the adult population spends most of their time. Not surprisingly, insomnia and work-related stress frequently co-occur and are interconnected [1]. Stress at work may lead, for example, to a lower sleep quality and an increase in insomnia symptoms [2-4]. In contrast, insomnia symptoms are negatively related to well-being at work and work pleasure [5]. Chronic insomnia has a 10% prevalence rate in Western industrialized countries [6,7], and 28% of Europeans suffer from work-related stress [8]. Moreover, approximately one-third of the population in Germany suffers from frequent sleep problems [9]. Insomnia and work stress are both associated with personal distress, diminished general functioning, increased absenteeism and presenteeism and, in turn, high personal and societal costs [9-15].

Numerous studies have investigated the relationship between work stress and health impairments such as insomnia. For example, Akerstedt [13] emphasized that a deficient recovery from work is one important risk factor for developing insomnia in people with work-related stress. Recovery can be defined as the process of psychophysiological unwinding after work, during which depleted resources are restored [16]. Based on the allostatic load model [17] and the effort-recovery model [18], this psychophysiological unwinding is crucial for mental and physical well-being. Currently, recovery is well-recognized as an essential daily process to balance the strains of work [19-21]. Cognitive detachment or ‘mentally switching off’ is a central determinant of recovery [1,16,22], and it has been found to mediate the relationship between work stress and insomnia complaints [23].

Cognitive detachment can be fostered by recreational activities [19]. Moreover, a deficient cognitive detachment in the form of worrying and rumination on work-related problems can be reduced by interventions such as Cognitive Behavioral Therapy (CBT) [24] or Metacognitive Therapy (MCT) [25]. Ample evidence supports the efficacy of Cognitive Behavioral Therapy for Insomnia (CBT-I). Several meta-analyses, including Morin, Culbert and Schwartz (1994), showed large effect sizes for sleep onset latency and moderate effect sizes for quality of sleep and total sleep time experienced by adults with chronic primary insomnia (for example, [26]). A meta-analysis by Smith *et al.* (2002) also illustrates that CBT-I is not only less risky with fewer adverse effects than pharmacological treatment, but also is more effective in the long term [27].

However, the gap between the need for CBT-I and its availability and implementation is large [28]. One reason for the deficiency in treatment might be that both patients and physicians often fail to recognize insomnia complaints as discrete symptoms requiring separate treatment. This neglect is problematic, given also that insomnia is a risk factor for other disorders such as depression [29,30].

Internet-based CBT interventions have been shown to be effective for several health problems such as depression, anxiety, chronic pain and problem drinking, with an overall effect size of d = .52 [31] which is comparable to face-to-face psychotherapy [32]. The effectiveness of iCBT-I has also been supported by strong evidence [33,34]. In a recent meta-analysis [33] significant effects were shown on sleep quality, sleep efficiency, number of awakenings, sleep onset latency and insomnia severity, ranging from moderate (for example, sleep quality) to large (insomnia severity) effect sizes. The underutilization of CBT-I could be potentially reduced by iCBT-I because of its several advantages such as its flexibility, broad availability and low threshold [35].

However, less is known about the efficacy of iCBT-I in people affected by high work stress and even less in specific working populations such as teachers. Studies consistently report that 10 to 35% of teachers suffer from stress-related health issues such as exhaustion, depressive or somatoform symptoms [36-39]. Moreover, teachers are more often affected by insomnia complaints than the general working population [40]. Research on the particular stressful situation of teachers has led to calls for better prevention of mental health problems in the educational sector of Germany [8]. Although the cost-effectiveness of internet-based CBT has shown promising results for several disorders [41] such as depression [42] or problem drinking [43], the cost-effectiveness of iCBT-I interventions has not yet been investigated.

The planned trial, to our knowledge, is the first to evaluate an iCBT-I intervention that investigates the efficacy and cost-effectiveness of an iCBT-I intervention tailored to a specific population affected by work-related strain.

In the proposed randomized controlled trial, a guided self-help iCBT-I intervention tailored to school teachers affected by clinical significant insomnia symptoms and high occupational strain will be evaluated with regard to the reduction of insomnia severity. We hypothesize that the intervention will lead to reduced insomnia complaints and will be superior to a wait-list control condition in terms of cost-effectiveness.

## Methods/Design

### Study design

This study is a randomized controlled trial with the following two groups: an Internet-based guided self-help intervention group (GET.ON Recovery) and a wait-list control group (WL). The study will comprise an effectiveness study and an economic evaluation in parallel. The economic evaluation will be conducted from a societal perspective. Assessments will be taken at post-treatment (eight weeks) and in a six-month follow-up. The study has been approved by the Medical Ethics Committee of the Philipps University of Marburg (Nr.: 2013-01K).

### Inclusion and exclusion criteria

Inclusion criteria are: being a primary, secondary or vocational school teacher above the age of 18, currently in work and with significant clinical insomnia symptoms as measured by a score ≥15 on the Insomnia Severity Index (ISI) [44] and work-related rumination as measured by a score ≥15 in the subscale ‘Cognitive Irritation’ of the Irritation Scale (IS) [45], access to the Internet, an email address and a telephone number. Subjects receiving psychological help for their sleep problems or showing suicidal ideation will be excluded from participation. People taking sleep medication will not be excluded from the study. To screen for possible suicidal ideation and intentions, we will use item 9 from the Beck Depression Inventory II (BDI II) [46] with scores of 0 (‘I don’t have any thoughts of harming myself’), 1 (‘I have thoughts of harming myself, but I would not carry them out’), 2 (‘I feel I would be better off dead’), and 3 (‘I would kill myself if I could’). Subjects scoring above the cut-off of 1 will be excluded from participation. We have a suicide protocol at place for those subjects who are excluded because of suicidal ideation. They will receive an email that refers them to their general practitioner and provides them with several telephone numbers and contacts that they can use to seek help.

### Procedure

**Figure 1:**
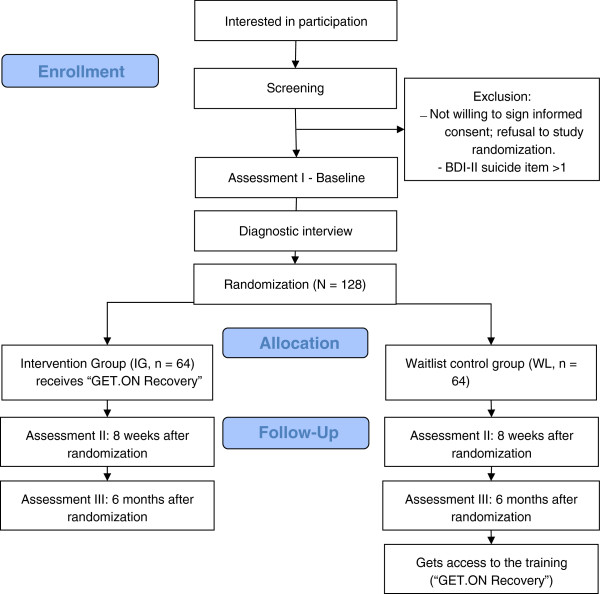
**Research procedure.** Subjects interested in participation receive online screening questionnaires. Eligible subjects then are asked to sign informed consent, fill in the baseline questionnaires and take part in a diagnostic interview via telephone. After that, subjects are randomized into either intervention or control group. Further assessments take place two and six months after randomization. Subjects of the control group get access to the intervention (self-help) following the last assessment.

Participants will be recruited during March to June 2013 through email distribution lists by the ministry of education in the German state of Nordrhein-Westfalen (NRW). Principals of primary, secondary and vocational schools are invited to circulate a flyer to their teaching staff. Interested teachers can apply for participation by an email to the primary study investigator (first author). The primary study investigator will provide interested teachers with a PDF containing additional detailed information and a request to complete an online screening questionnaire, which will be available on the same homepage that will contain all other study questionnaires and the online training. Teachers will obtain access to the homepage by using their email address and a self-chosen password. We will provide eligible participants with an ID number and then ask them to a) provide informed consent, b) complete baseline questionnaires (including a 7-day sleep diary) and c) participate in a diagnostic interview via telephone. The diagnostic interview will take about 20 minutes and include sections from the Structured Clinical Interview for DSM-IV (SKID-I) [47] and the Structured Interview for Sleep Disorders (SIS-D) [48]. The diagnostic interview will not be used as an inclusion or exclusion measure but will provide diagnostic data for primary insomnia, major depression and generalized anxiety disorder. After the diagnostic interview, participants will be randomized. Assessments will occur at baseline, before randomization (T0), eight weeks post-treatment (T1) and six months follow-up (T2). We chose eight weeks for T1 as a post-treatment measure to ensure that all participants will have completed the six week training. Figure 1 illustrates an overview of the procedures the participants will undergo.

### Randomization

A computer program will automatically assign a 0 or 1 to each ID number, indicating the allocation to either the intervention or a waiting list. This random assignment is made in a 1:1 ratio.

### Sample size

The sample size will be based on the difference between experimental and control group at post-test on the primary outcome measure (insomnia severity). We expect a difference of Cohen’s d = .50. Given an alpha of .05 and 1 - beta = .80 in a two-tailed test, we will need 64 subjects in each condition with a total sample size of N = 128. Based on a recent meta-analysis on the efficacy of online insomnia trials [33], we could expect a higher effect size (d = .86) for our primary outcome. But our target group differs from the group assessed in the meta-analysis, thus we chose a rather conservative estimate to reduce the chance of an under-powered study.

### Intervention

The online recovery training for better sleep (GET.ON Recovery) is a newly developed iCBT-I training, which uses well-established CBT-I methods such as sleep restriction, stimulus control, sleep hygiene and cognitive interventions [49]. These methods are supplemented by techniques from behavioral activation, MCT [50] and gratitude research [51].

The theoretical assumption underpinning GET.ON Recovery is that the participants’ insomnia symptoms are caused and maintained by an impaired or even missing recovery from their daily work-related effort. Thus, symptoms will improve if their recovery is enhanced during the night (better sleep through sleep hygiene, stimulus control and sleep restriction) and at the weekend (better cognitive detachment from work-related problems through recreational activities, and through worry and rumination techniques) [26,52].

The intervention consists of six one-week sessions. In the first session, participants will receive information about recovery (that is, how sleep, cognitive detachment and recreational activities are interconnected), and they will be educated on sleep hygiene. In session two, participants will learn why stimulus control and sleep restriction are effective methods to overcome sleep problems. Participants will be asked to plan the first step in sleep restriction and accordingly reschedule their sleep for the following week. In session three, participants will review their progress on sleep restriction and sleep hygiene and then schedule their sleep for the next week according to their sleep restriction plan. Then they will be presented with ‘boundary tactics’ [53], which are practical behaviors that help to clearly distinguish work and private life. The session will end with an exercise on the gratitude journal, a well-studied method on how to end the day and to improve sleep [51]. In sessions four and five, participants will be provided with psycho-education about worrying and rumination and how it influences sleep. Different exercises will be presented, all aimed at fostering the ability to become more cognitively detached from work-related problems, and in turn reduce worrying and rumination. The fifth session is based on MCT [54], which is grounded in the assumption that enduring attention on thoughts can further increase their frequency and intensity. Participants will learn two MCT exercises: detached mindfulness and attention training.

In every session, homework will be given to help participants actively plan and implement recreational activities into daily life according to the behavioral activation approach. Additionally, participants will be asked to choose one exercise from the session to apply throughout the forthcoming week. The intervention will conclude with a ‘relapse-prevention’ session (session six) that summarizes what will have happened in the preceding five sessions. This session will provide an opportunity for participants to reflect and elaborate on their progress and future goals. Additionally, participants will choose exercises from the preceding five sessions that they believe will help prevent relapse (when sleep gets significantly worse).

### Support

The participants will be supported by trained coaches according to a manual written by the first author. The support will consist of the general availability of the coach to answer any questions that might occur, reminders sent by the coach when a patient has not completed a module in seven days, and brief feedback provided by the coach on completed online exercises (within the module) and on the completion of the homework assignments. The coach will write the feedbacks via a messaging system on the training homepage after the respective participant will have finished a module.

The total amount of time spent on each participant will be about three hours for the total duration of the intervention. The coaches will be supervised (first author).

### Experimental condition

Participants in the intervention group will receive GET.ON Recovery after randomization. Participants in the control group receive GET.ON Recovery six months after randomization. Subjects of the control group will not be supported with feedback or reminders, but the primary study investigator will be available for questions concerning the content of the intervention or for technical problems.

### Instruments

Table 1 provides an overview of the applied instruments at each point in time. We expect participants to need approximately 30 minutes to complete the questionnaires at one point in time.

**Table 1:** Overview of instruments per time of measurement (T0, T1, T2)

	**Number of items**	**Screening**	**T0**	**T1**	**T2**
			**(baseline)**	**(8 weeks)**	**(6 months)**
Socio-demographic data	20	x	-	-	-
Insomnia severity (ISI)	7	x	x	x	x
Work stress (IS, Subscale CI)	3	x	x	x	x
Sleep effort (GSES)	7	-	x	x	x
Sleep diary	10	-	x	x	-
Adherence to sleep hygiene (SHI)	13	-	x	x	x
Recuperation in sleep (items of SF-B)	7	-	x	x	x
Sleep quality (item of PSQI)	1	-	x	x	x
Effort reward imbalance (ERI-S)	10	-	x	-	-
Depressive symptoms (ADS)	20	-	x	x	x
Worrying (PSWQ-PW, ultra short)	3	-	x	x	x
Recovery experiences (REQ)	16	-	x	x	x
Recovery activities (ReaQ)	21	-	x	x	x
Quality of life (SF-12)	12	-	x	x	x
Presenteeism, absenteeism and health care utilization (TicP)	Depends	-	x	-	x
Teacher self-efficacy (Teacher self-efficacy scale)	10	-	-	-	x
Work engagement (UWES)	9	-	x	-	-
User satisfaction	8	-	-	x	-

*ADS* Allgemeine Depressionsskala, *CI* Cognitive Irritation, *ERI-S* effort-reward imbalance – short version, *GSES* Glasgow Sleep Effort Scale, *IS* Irritation Scale, *PSQI* Pittsburgh Sleep Quality Index, *PSWQ-PW* Penn State Worry Questionnaire – Past Week, *ReaQ* Recovery experience and activities questionnaire, *REQ* Recovery Experience Questionnaire, *SF-12* Short Form Health Survey – 12 items, *SF-B* Schlaffragebogen B, *SHI* Sleep Hygiene Index, *TicP* Trimbos/iMTA questionnaire for costs associated with Psychiatric illness, *UWES* Utrecht Work Engagement Scale.

#### Screening

The ISI [44], the ‘Cognitive Irritation’ subscale of the IS [45], and item 9 of the BDI II [46] will be administered as screening measures.

#### Primary outcome measure

The primary outcome measure is insomnia severity (ISI).

##### Insomnia severity

Insomnia severity will be assessed by the Insomnia Severity Index (ISI) [44,55]. This 7-item instrument has been developed by Morin, is validated, also as a web-based measure [56] and frequently used in iCBT-I research (for example, [57]). The responses are on a 5-point Likert scale and the sum score, ranging from 0 to 28, is an index of overall insomnia severity, with 0 to 7 indicating no clinically significant insomnia, 8 to 14 sub-threshold insomnia, 15 to 21 clinical insomnia (moderate severity), and 22 to 28 clinical insomnia (severe). We will use the cut-off score of 14, because a score greater than 14 indicates clinical insomnia. We use the German version (Pillmann, F., unpublished).

#### Secondary outcome measures

**Demographics** A self-designed questionnaire will be used to collect participants’ demographic data, such as living situation, type of school they work in and age.

##### Sleep effort

We will measure sleep effort with the Glasgow Sleep Effort Scale (GSES). This 7-item scale (for example, ‘I put too much effort into sleeping when it should come naturally’) is answered on a 3-point Likert scale (very much, to some extent, not at all). The scale has been validated with a reported internal consistency of .77 [58].

##### Sleep diary

Participants will complete an online sleep diary for seven days, both at T0 and T1. In the diary, participants will record the time at which they leave their bed each morning, their previous evening bedtime and the total hours of sleep. Sleep efficiency (SE) and total sleep time (TST) will be computed as outcome measures from the diary data. Additionally, the diary will also contain data, such as information regarding sleep quality, worrying before going to bed, sleep effort and recreational activities in the previous day. Participants will be encouraged to use this diary also in the course of the intervention.

##### Sleep hygiene

The Sleep Hygiene Index [59] is a validated instrument comprising 13 items that assess the frequency of sleep-impaired behaviors during the previous 14 days (for example, ‘I think, plan, or worry when I am in bed’) and a 5-point Likert scale (0 = never; 4 = always). We will recode the sum score so that higher scores indicate a better sleep hygiene.

##### Recuperation in sleep

Recuperation in sleep can be assessed by two items from a well-validated questionnaire [60] that has been recommended as an outcome measure by the German Society of Sleep Medicine (DGSM). Participants rate a) if they needed some extra time to ‘get going’ in the morning and b) how they felt after awakening during the previous 14 days according to seven feelings (for example, tired, relaxed). Both items are answered on a 5-point Likert scale (never, almost never, sometimes, often, always).

##### Sleep quality

We will assess sleep quality with one single item derived from the Pittsburgh Sleep Quality Index (PSQI) [61]. The answer is given on a 4-point Likert scale (‘How do you evaluate the quality of this week’s sleep?’ 1 = very good; 4 = very poor). We will recode the item so that higher scores indicate better sleep quality.

##### Work stress

We will assess work stress with a validated 10-item short version of the Effort-Reward-Imbalance questionnaire (ERI-S) [62]. All items (for example, ‘Considering all my efforts and achievements, I receive the respect and prestige I deserve at work’) are answered on a 4-point Likert scale (1 = strongly agree; 4 = strongly disagree). By using the two subscales, ‘effort’ and ‘reward’, a score for ‘effort-reward imbalance’ can be computed. The ERI-S is a well-established measure for work stress, and its psychometric quality is satisfactory to good [62].

##### Depressive symptoms

The Allgemeine Depressions-Skala (ADS) [63] is the German adaptation of the Centre for Epidemiological Studies Depression Scale (CES-D) developed at the National Institute for Mental Health. It is a frequently and commonly used self-report instrument measuring depressive symptoms in the week preceding the assessment. The ADS consists of 20 items answered on a 4-point Likert scale. The total score ranges between 0 and 60. Internal consistency is reported to be α = .89, split-half reliability is *r* = .91 [63].

##### Worrying

We will use a 3-item short version of the Penn State Worry Questionnaire (PSWQ) [64,65] and adapt it as a past-week version according to the respective three German items of the Penn State Worry Questionnaire-Past Week (PSWQ, PW [66]. The questionnaire assesses pathological worry during the past week and is answered on a 7-point Likert scale. Internal consistency of the English ultra-brief version of the PSWQ is reported to be α = .85, which is comparable to the internal consistency of the English PSWQ and the German PSWQ-PW version.

##### Work-related rumination

In conformance with Berset [23], we will measure work-related rumination with the ‘Cognitive Irritation’ subscale of the Irritation Scale (IS, [45]. Internal consistency is reported to be α = .86. A score of 15 or higher indicates an above-average level of rumination (Stanine ≥7) [45].

##### Recovery experiences

The recovery experience questionnaire (REQ [67], is a validated 16-item measure answered on a 5-point Likert scale that assesses recuperation and unwinding from work. The items load on four factors that represent four different recovery experiences: psychological detachment (α = .85), relaxation (α = .85), mastery (α = .85) and control (α = .85). A sum score can be computed for each scale.

##### Recovery activities

The Recreation experience and activity Questionnaire, (ReaQ) by Lehr (DL, paper submitted) comprises 21 items that assess the frequency of several recreational activities during the past week. Answer categories include ‘never’, ‘one time’, ‘two times’, ‘three times’ or ‘at least 4 times’. Internal consistency of the questionnaire is α = .83.

##### Teacher self-efficacy

Self-efficacy will be measured by the Teacher Self-Efficacy Scale [68], a 10-item self-rating instrument that has been shown to have good reliability and validity and to have potential for predicting work-related stress symptoms [69].

##### Work engagement

Work engagement is assessed by the Utrecht Work Engagement Scale (UWES, German version) [70]. Its 17 items represent three subscales: vigor, dedication and absorption. Internal consistency is α = .91 for the total score.

##### User satisfaction

We will assess user satisfaction by a self-designed questionnaire based on the ‘Satisfaction with Psychotherapy’ Questionnaire (ZUF-8) [71] and the German version of the Client Satisfaction Questionnaire (CSQ-8) [72]. We will also assess satisfaction before the beginning of each module by asking participants how useful and helpful the information from the previous module appeared to them.

##### Quality of life

Improvement of health-related quality of life (HRQL) is measured by the well-established short version (SF-12) [73] of the Short Form 36 Health Survey Questionnaire (SF-36) [73]. This self-report measure is a generic, 12-item, multi-purpose health survey, with eight scales (Physical Functioning, Role-Physical, Bodily Pain, General Health, Vitality, Social Functioning, Role-Emotional, Mental Health) and two summary measures indicating quality of life with regard to mental health and quality of life with regard to physical health. Most of the items are answered on a 3-, 5- or 6-point Likert scale.

#### Economic evaluation

**Quality-adjusted life years (QALYs)** QALYs will be obtained from the SF-6D [73]. The SF-6D contains six dimensions (each with between 2 and 5 levels) and includes 7 items of the SF-12. The SF-6D generates 7500 different health states. Utility values will be derived using Brazier’s algorithm [74,75].

##### Presenteeism, absenteeism and health care utilization

We adapted the Trimbos/iMTA questionnaire for costs associated with Psychiatric Illness (TiC-P) [76] due to insomnia and occupational stress in German teachers.

### Statistical analysis

The analyses will be conducted according to the CONSORT guidelines. All analyses will be based on the intention-to-treat sample. Missing values will be imputed by state of the art imputation methods.

#### Treatment efficacy

We will perform repeated measures ANOVAs to compare the intervention group and the waitlist control group on the primary and secondary outcome measures. Cohen’s d will be used for the between group effect size, calculated [77]. Apart from statistical significance tests, we will also look at the clinical significance of our data by detecting reliable changes and recovery rates using the method of Jacobson and Truax [78].

#### Diary data

Sleep efficiency (SE) and total sleep time (TST) will be recorded in a sleep diary at pre- (T0) and post-treatment (T1), seven times in the course of seven days. We will compare SE and TST means in a repeated-measures ANOVA to evaluate treatment efficacy (see above). Diary data participants collect over the course of the intervention will be explored using multilevel modeling procedures.

#### Economic analyses

We will calculate direct and indirect costs and look at cost-effectiveness and cost-utility ratios to assess the economic impact of mental illness due to insomnia and occupational stress. A comparison will be made between the intervention and control groups on incremental costs and incremental effects. The incremental cost-effectiveness ratio (ICER) will be calculated as follows: ICER = (Δ costs/Δ effects) where Δ costs represents the difference in annual mean costs between intervention and control group, and Δ effects represents the difference in annual mean effects between the two groups. Bootstrapping with 5,000 iterations will be used to test the robustness of the ICER and quantify the uncertainty around the ratios, which will be plotted in a cost-effectiveness plane. The bootstrapped ICERs will also be presented in a cost-effective acceptability curve displaying the probability that the intervention is cost-effective for a wide range of willingness-to-pay ceilings. To test the robustness of the base-case findings, a multi-way sensitivity analysis will be performed. Several assumptions made in the base-case scenario (that is, concerning costs and volumes) will be changed to assess their impact on the ICER.

## Discussion

Existing iCBT-I interventions have been demonstrated to be effective for reducing insomnia complaints [33]. However, people suffering from chronic insomnia mainly receive generalized CBT-I [33], regardless of the specificity of the target group or the context in which insomnia symptoms occur. People with insomnia complaints who are highly affected by work-related stress might need a tailored treatment because their insomnia symptoms are developed and maintained by respective psychological strain such as deficient cognitive detachment from work and work-related rumination [4,16,22,23]. We therefore developed an intervention that advances methods that might promote better recovery from work (better cognitive detachment from work-related problems) than classic CBT-I elements such as sleep restriction, stimulus control and sleep hygiene. That is, the method not only targets general insomnia symptoms but also the specific context in which they appear and are prolonged. Additionally, the cost-effectiveness of iCBT-I has not yet been investigated. Given the association of insomnia and work-related stress with high absenteeism and presenteeism and thus, with high economic costs [9-15], cost-effective treatments are needed. We included an economic evaluation in our study to address this need.

In the proposed trial, we aim to evaluate a newly developed guided iCBT-I intervention tailored to the teaching profession, which is often referred to as being especially affected by stress-related health symptoms, such as insomnia and depression. We will evaluate the training for its efficacy and cost-effectiveness.

This study will have three major strengths. First, compared to a general population of employees from different disciplines, the occupational demands are relatively homogenous within the population of teachers [40]. Thus, the present study is expected to be characterized by high internal validity.

Second, we will look at treatment efficacy and mechanisms of change from a variety of angles. Apart from classic sleep-related measures such as insomnia severity, sleep quality or sleep efficiency, we will assess worrying and work-related rumination because perseverative cognitions are expected to play a mediating role between stress and insomnia. We will also focus on sleep effort, a concept that is known to play a crucial role in the etiology and persistence of insomnia [53]. Yet, to our knowledge, with only one exception [79], no CBT-I or iCBT-I trial has used the measure as primary or secondary outcome. Overall, our measures will address three essential elements of recovery which are crucial for general mental well-being: sleep, cognitive detachment and recreational activities [13].

Third, we will opt for a conservative approach for computing our sample size by including more participants than a recent meta-analysis [33] would indicate. We used a more conservative estimate (d = .50) than the effect size reported in the meta-analysis (d = .86) for following three reasons: 1) The meta-analysis included only two trials with regard to the insomnia severity index, 2) our training is not a generic iCBT-I intervention but instead follows a new concept adapting iCBT-I for people affected by occupational strain, and 3) with a sample size calculation based on the higher effect size, according to the same meta-analysis, most analyses of secondary outcomes such as other sleep measures would be under-powered.

The study will have some limitations, primarily with regard to generalizability. Results might only be valid for teachers and occupational groups with comparable demographic and job-related characteristics such as fuzzy boundaries between work and non-work life or working in the afternoon and the evening at the home office [38]. Also, given that 70.4% of teachers are female [80], we expect that our sample will be analogously ‘unbalanced’, restricting the validity for male teachers. Finally, given the focus of our investigation on the efficacy of our intervention as a whole, we cannot make conclusions about the extent that newly developed intervention components (for example, session 5 that contains methods from MCT) add to sessions containing classic CBT-I methods.

Overall, to overcome the gap between need and actual implementation of CBT-I, (cost)-effective treatments for insomnia are needed that are highly accessible for as many people as possible [28]. A stepped-care approach to treatment delivery is one possibility to achieve this goal [81]. According to the stepped-care model, interventions low in cost and intensity are delivered first. If people do not respond to those treatments, more expensive and intensive interventions are offered [82]. If the proposed trial shows that our training could be (cost)-effective, it can potentially serve as a first low-intensity intervention in a stepped-care approach [81], especially for (a constantly increasing number of) people affected by work stress.

## Trial status

The study is currently ongoing. Recruitment started at the end of February 2013 and will be finished June 2013.

## Abbreviations

ADS: Allgemeine Depressionsskala; CBT: Cognitive behavioral therapy; CBT-I: Cognitive behavioral therapy for insomnia; CI: Cognitive irritation; CSQ-8: Client satisfaction questionnaire; DGSM: German society of sleep medicine; ERI-S: Effort-reward imbalance - short version; GSES: Glasgow sleep effort scale; iCBT-I: Internet-based cognitive behavioral therapy for insomnia; ICER: The incremental cost-effectiveness ratio; IG: Intervention group; IS: Irritation scale; ISI: Insomnia severity index; MCT: Metacognitive therapy; PSQI: Pittsburgh sleep quality index; PSQW-PW: Penn State Worry Questionnaire - past week; QALYs: Quality-adjusted life years; REQ: Recovery experience questionnaire; SE: Sleep efficiency; SF-B: Schlaffragebogen-B; SF-12: Short form health survey - 12 items; SHI: Sleep hygiene index; TicP: Trimbos/iMTA questionnaire for costs associated with psychiatric illness; TST: Total sleep time; UWES: Utrecht work engagement scale; WL: Waitlist control group.

## Competing interests

Professor Berking and Professor Sieland are minority shareholders of Minddistrict GmbH, which provides the online platform for the training.

## Authors’ contributions

All authors contributed to the design of this study. HT, DL DE and BS developed the iCBT-I intervention (‘GET.ON Recovery’), HT drafted the manuscript, HR supervised the writing process. All authors contributed to the further writing of the manuscript. All authors read and approved the final manuscript.
